# Supplementary material for: A cluster-randomized trial of client and provider directed financial interventions to align incentives with appropriate case management in private medicine retailers: Results of the TESTsmART trial in Lagos, Nigeria
Source: PLOS Glob Public Health. 2024 Jul 2;4(7):e0002938. doi: 10.1371/journal.pgph.0002938 (PMC11218985; doi:10.1371/journal.pgph.0002938)
Supplement: S2 Text — Table 1: Secondary Outcomes- Sample characteristics by intervention arm and mRDT use Table 2: Adherence by client gender and test result, among mRDT purchasers Table 3: Proportion who purchased antibiotics, overall and by subgroups (post-design change only) Table 4: Injections summary. (DOCX) [file pgph.0002938.s003.docx]

**S2 Text**

Table 1: Secondary Outcomes- Sample characteristics by intervention arm and mRDT* use.

Table 2: Adherence by client gender and test result, among mRDT purchasers.

Table 3: Proportion who purchased antibiotic, overall and by subgroups (post-design change only).

Table 4: Injections summary.

**Table S1 Secondary Outcomes- Sample characteristics by intervention arm and mRDT* use.**

|  | **mRDT, N = 443** | | | **No mRDT, N = 1757** | | |
| --- | --- | --- | --- | --- | --- | --- |
| **Characteristic** | **Control** | **CDPD** | **Overall** | **Control** | **CDPD** | **Overall** |
| Interviewees, N | 226 | 217 | 443 | 874 | 883 | 1,757 |
| Missing | 0 | 0 | 0 | 0 | 0 | 0 |
| Respondent, n (%) |  |  |  |  |  |  |
| Adult client | 176 (78%) | 187 (86%) | 363 (82%) | 749 (86%) | 700 (79%) | 1,449 (83%) |
| Guardian of child client | 50 (22%) | 30 (14%) | 80 (18%) | 125 (14%) | 182 (21%) | 307 (17%) |
| Missing | 0 | 0 | 0 | 0 | 1 | 1 |
| Respondent age, n (%) |  |  |  |  |  |  |
| 18-25 | 41 (18%) | 34 (16%) | 75 (17%) | 149 (17%) | 143 (16%) | 292 (17%) |
| 26-39 | 106 (47%) | 109 (50%) | 215 (49%) | 399 (46%) | 448 (51%) | 847 (48%) |
| 40-59 | 72 (32%) | 56 (26%) | 128 (29%) | 295 (34%) | 256 (29%) | 551 (31%) |
| 60-79 | 7 (3.1%) | 15 (6.9%) | 22 (5.0%) | 28 (3.2%) | 28 (3.2%) | 56 (3.2%) |
| 80 and above | 0 (0%) | 2 (0.9%) | 2 (0.5%) | 3 (0.3%) | 1 (0.1%) | 4 (0.2%) |
| Missing | 0 | 1 | 1 | 0 | 7 | 7 |
| Client gender, n (%) |  |  |  |  |  |  |
| Male | 98 (43%) | 97 (45%) | 195 (44%) | 494 (57%) | 445 (50%) | 939 (53%) |
| Female | 128 (57%) | 120 (55%) | 248 (56%) | 380 (43%) | 438 (50%) | 818 (47%) |
| Missing | 0 | 0 | 0 | 0 | 0 | 0 |
| Respondent gender, n (%) |  |  |  |  |  |  |
| Male | 88 (39%) | 93 (43%) | 181 (41%) | 465 (53%) | 403 (46%) | 868 (49%) |
| Female | 138 (61%) | 124 (57%) | 262 (59%) | 409 (47%) | 479 (54%) | 888 (51%) |
| Missing | 0 | 0 | 0 | 0 | 1 | 1 |
| Adult client age, n (%) |  |  |  |  |  |  |
| 18-25 | 38 (22%) | 34 (18%) | 72 (20%) | 140 (19%) | 139 (20%) | 279 (19%) |
| 26-39 | 76 (43%) | 92 (49%) | 168 (46%) | 327 (44%) | 320 (46%) | 647 (45%) |
| 40-59 | 55 (31%) | 43 (23%) | 98 (27%) | 252 (34%) | 215 (31%) | 467 (32%) |
| 60-79 | 7 (4.0%) | 15 (8.1%) | 22 (6.1%) | 27 (3.6%) | 25 (3.6%) | 52 (3.6%) |
| 80 and above | 0 (0%) | 2 (1.1%) | 2 (0.6%) | 3 (0.4%) | 1 (0.1%) | 4 (0.3%) |
| Missing | 0 | 1 | 1 | 0 | 0 | 0 |
| Adult client gender, n (%) |  |  |  |  |  |  |
| Male | 76 (43%) | 82 (44%) | 158 (44%) | 438 (58%) | 357 (51%) | 795 (55%) |
| Female | 100 (57%) | 105 (56%) | 205 (56%) | 311 (42%) | 343 (49%) | 654 (45%) |
| Missing | 0 | 0 | 0 | 0 | 0 | 0 |
| Child client age |  |  |  |  |  |  |
| Mean (SD) | 5.9 (3.7) | 7.2 (4.1) | 6.4 (3.9) | 6.7 (4.3) | 5.9 (4.3) | 6.2 (4.3) |
| Median [IQR] | 5.0 [3.0, 8.0] | 6.5 [3.2, 10.0] | 5.0 [3.0, 10.0] | 6.0 [3.0, 10.0] | 4.9 [2.0, 9.0] | 5.0 [2.5, 10.0] |
| Range | 1.0, 15.0 | 2.0, 15.0 | 1.0, 15.0 | 1.0, 17.0 | 1.0, 17.0 | 1.0, 17.0 |
| Missing | 0 | 0 | 0 | 1 | 0 | 1 |
| Child client gender, n (%) |  |  |  |  |  |  |
| Male | 22 (44%) | 15 (50%) | 37 (46%) | 56 (45%) | 87 (48%) | 143 (47%) |
| Female | 28 (56%) | 15 (50%) | 43 (54%) | 69 (55%) | 95 (52%) | 164 (53%) |
| Missing | 0 | 0 | 0 | 0 | 0 | 0 |
| Education level, n (%) |  |  |  |  |  |  |
| None | 11 (5.4%) | 5 (2.4%) | 16 (3.9%) | 42 (5.3%) | 31 (4.1%) | 73 (4.7%) |
| Primary | 12 (5.9%) | 11 (5.3%) | 23 (5.6%) | 58 (7.3%) | 26 (3.4%) | 84 (5.4%) |
| Secondary | 79 (39%) | 113 (55%) | 192 (47%) | 393 (49%) | 330 (44%) | 723 (46%) |
| University | 55 (27%) | 38 (18%) | 93 (23%) | 141 (18%) | 217 (29%) | 358 (23%) |
| Polytechnic | 46 (23%) | 39 (19%) | 85 (21%) | 165 (21%) | 154 (20%) | 319 (20%) |
| Missing | 23 | 11 | 34 | 75 | 125 | 200 |
| Wealth index (quintile), n (%) |  |  |  |  |  |  |
| 0 to 20^th^ | 38 (19%) | 40 (21%) | 78 (20%) | 207 (27%) | 157 (21%) | 364 (24%) |
| >20.0 to 40th | 30 (15%) | 38 (20%) | 68 (18%) | 136 (18%) | 109 (15%) | 245 (16%) |
| >40.0 to 60th | 57 (29%) | 36 (19%) | 93 (24%) | 196 (26%) | 169 (23%) | 365 (25%) |
| >60.0 to 80th | 40 (20%) | 37 (20%) | 77 (20%) | 113 (15%) | 133 (18%) | 246 (17%) |
| >80.0 | 35 (18%) | 37 (20%) | 72 (19%) | 102 (14%) | 164 (22%) | 266 (18%) |
| Missing | 26 | 29 | 55 | 120 | 151 | 271 |
| Time Period, n (%) |  |  |  |  |  |  |
| Feb-May 2022 | 45 (20%) | 43 (20%) | 88 (20%) | 181 (21%) | 164 (19%) | 345 (20%) |
| Jun-Aug 2022 | 58 (26%) | 40 (18%) | 98 (22%) | 183 (21%) | 266 (30%) | 449 (26%) |
| Sep-Nov 2022 | 90 (40%) | 73 (34%) | 163 (37%) | 268 (31%) | 259 (29%) | 527 (30%) |
| Dec 2022-Feb 2023 | 33 (15%) | 61 (28%) | 94 (21%) | 242 (28%) | 194 (22%) | 436 (25%) |
| Missing | 0 | 0 | 0 | 0 | 0 | 0 |
| *Only mRDTs conducted at the shop are included | | | | | | |

**Table S2: Adherence by client gender and test result, among mRDT purchasers.**

|  | **Study Arm** | |  |
| --- | --- | --- | --- |
| **Characteristic** | **Male^1^** | **Female^1^** | **Overall^1^** |
| mRDT tested clients whose treatment adhered to test results | 116 / 195 (59%) | 169 / 248 (68%) | 285 / 443 (64%) |
| Positive mRDT clients whose treatment adhered to test results | 23 / 26 (88%) | 23 / 30 (77%) | 46 / 56 (82%) |
| Negative mRDT clients whose treatment adhered to test results | 93 / 162 (57%) | 146 / 213 (69%) | 239 / 375 (64%) |
| **^1^n / N (%)** | | | |
|  | | | |

**Table S3: Proportion who purchased antibiotics, overall and by subgroups (post-design change only).**

|  |  |
| --- | --- |
|  | **Purchased antibiotic** |
| Overall |  |
| All interviewees | 597/2,205 (27%) |
| Client gender |  |
| Male | 287/1,136 (25%) |
| Female | 310/1,068 (29%) |
| Missing | 0/1 (0%) |
| Client age |  |
| Under 5 years | 41/176 (23%) |
| 5-17 years | 72/212 (34%) |
| 18+ years | 483/1,815 (27%) |
| Missing | 1/2 (50%) |
| Respondent gender |  |
| Male | 271/1,051 (26%) |
| Female | 325/1,152 (28%) |
| Missing | 1/2 (50%) |
| Respondent age |  |
| 18-25 | 87/367 (24%) |
| 26-39 | 300/1,065 (28%) |
| 40-59 | 190/679 (28%) |
| 60-79 | 19/78 (24%) |
| 80 and above | 0/6 (0%) |
| Missing | 1/10 (10%) |
| Education level |  |
| None | 29/89 (33%) |
| Primary | 26/107 (24%) |
| Secondary | 243/915 (27%) |
| University | 120/451 (27%) |
| Polytechnic | 105/404 (26%) |
| Missing | 74/239 (31%) |
| Wealth index (quintile) |  |
| 0 to 20^th^ | 114/442 (26%) |
| >20.0 to 40^th^ | 82/313 (26%) |
| >40.0 to 60^th^ | 141/458 (31%) |
| >60.0 to 80^th^ | 80/323 (25%) |
| >80.0 | 82/338 (24%) |
| Missing | 98/331 (30%) |
| mRDT results |  |
| Positive | 20/56 (36%) |
| Negative | 66/375 (18%) |
| No mRDT | 506/1,757 (29%) |
| Missing | 5/17 (29%) |
| ACT purchasing |  |
| ACT | 386/1,184 (33%) |
| No AM | 77/564 (14%) |
| Other (non-ACT) AM | 134/457 (29%) |

**Table S4: Injections summary.**

|  | **Test status** | | | **Study Arm** | | |
| --- | --- | --- | --- | --- | --- | --- |
| **Characteristic** | **No test, N = 1,703^1^** | **Positive test (any source), N = 127^1^** | **Negative test (shop), N = 375^1^** | **Control, N = 1,101^1^** | **CDPD, N = 1,104^1^** | **Overall^1^** |
| Any injection reported | 25 (1.5%) | 6 (4.7%) | 4 (1.1%) | 18 (1.6%) | 17 (1.5%) | 35 (1.6%) |
| Injection type |  |  |  |  |  |  |
| Identified as artemisinin injection | 22 (88%) | 3 (50%) | 2 (50%) | 15 (83%) | 12 (71%) | 27 (77%) |
| Identified as 'malaria injection' or 'antimalarial injection' or quinine injection | 2 (8.0%) | 3 (50%) | 2 (50%) | 3 (17%) | 4 (24%) | 7 (20%) |
| Painkiller or antibiotic injection | 1 (4.0%) | 0 (0%) | 0 (0%) | 0 (0%) | 1 (5.9%) | 1 (2.9%) |
| Unspecified injection | 0 (0%) | 0 (0%) | 0 (0%) | 0 (0%) | 0 (0%) | 0 (0%) |
| **^1^n (%)** | | | | | | |
